# Ghost Detection and Removal Based on Two-Layer Background Model and Histogram Similarity

**DOI:** 10.3390/s20164558

**Published:** 2020-08-14

**Authors:** Yiping Xu, Hongbing Ji, Wenbo Zhang

**Affiliations:** School of Electronic Engineering, Xidian University, Xi’an 710071, China; ypxu1208@163.com (Y.X.); zwbsoul@163.com (W.Z.)

**Keywords:** motion detection, background subtraction, ghosts, histogram similarity, local binary similarity pattern (LBSP), sample-based background model

## Abstract

Detecting and removing ghosts is an important challenge for moving object detection because ghosts will remain forever once formed, leading to the overall detection performance degradation. To deal with this issue, we first classified the ghosts into two categories according to the way they were formed. Then, the sample-based two-layer background model and histogram similarity of ghost areas were proposed to detect and remove the two types of ghosts, respectively. Furthermore, three important parameters in the two-layer model, i.e., the distance threshold, similarity threshold of local binary similarity pattern (LBSP), and time sub-sampling factor, were automatically determined by the spatial-temporal information of each pixel for adapting to the scene change rapidly. The experimental results on the CDnet 2014 dataset demonstrated that our proposed algorithm not only effectively eliminated ghost areas, but was also superior to the state-of-the-art approaches in terms of the overall performance.

## 1. Introduction

With the widespread application of surveillance cameras, a huge amount of video data is generated every day. Methods to automatically and quickly analyze information of interest from a video sequence has been extensively studied for decades [1,2,3,4,5]. Among these research works, change detection is a fundamental first step for higher-level computer vision applications such as video surveillance, pedestrian and vehicle tracking, and anomaly behavior recognition. Background subtraction (BS) is one of the most widely used techniques in change detection, and its performance depends mainly on the background modeling methods. To date, the popular background modeling methods can be classified into the following four categories: GMM-based [6,7,8,9], sample-based [10,11,12,13], clustering-based [14,15,16,17], and artificial neural network-based [18,19,20,21,22]. Each model has its own advantages and disadvantages. For example, the GMM-based model can handle the multimodal distribution problem, but the background pixel does not always follow Gaussian distribution and the difficulty of parameter estimation. The sample-based model shows the superiority in speed, yet it cannot efficiently address dynamic background and noise. The clustering-based model is robust to the noise, but only has good results for scenarios without substantial background changes. The artificial neural network-based model can obtain good performance, whereas this method requires previous training or a manual intervention.

In fact, robust change detection in real surveillance applications still faces great challenges in complex outdoor scenes [23,24,25], such as illumination changes, camera motion, ghost removal, camouflaged object detection, dynamic background suppression, and so on. Especially, ghost detection and removal are rarely discussed in the existing methods. This can be attributed to the following two difficulties. First of all, the initialization method of the background model is relatively simple. Due to the lack of prior knowledge of moving objects, the background model can only be initialized using the feature of the first few video frames in order to satisfy real time. Therefore, objects exist in the first frame will be treated as the background. Second, ghosts and long-term static foreground share many similar features, and it is difficult to eliminate ghosts and reserve static foreground simultaneously. For example, Cucchiara et al. [26] used the average optical flow of a moving object blob to discriminate between moving object and ghosts. However, this method leads to a high false negative rate on the static or uniform moving object because of a near-to-zero average optical flow. Wang et al. [7] differentiated stopped objects from revealed background by removed objects (ghosts) using the edge similarity between current image and foreground mask. This method relies on the accuracy of the edge detection. Actually, it is difficult to obtain accurate edge in complex scenarios because of the shadow, noise, and camouflage. The literatures [11,12,13,16,23] accelerated the elimination of ghosts by the spatial diffusion mechanism because of high similarities between the ghost region and the surrounding background. However, the camouflaged object is incorporated into the background.

**Figure 1:**
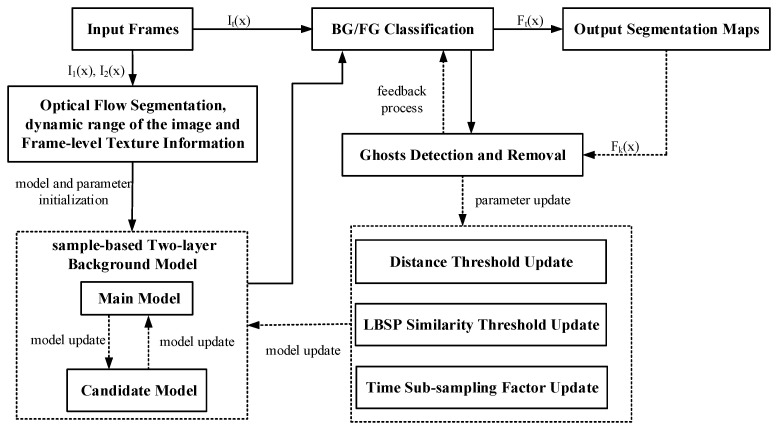
Block diagram of GhostDeReBS. The solid arrows indicate the processing flow of our systems for each frame; the dashed arrows indicate the update and feedback steps of our model.

Generally, ghosts can occur in the following two cases: (1) The foreground object that remains motionless for a long time is incorporated into the background. Later on, the foreground object is removed, and the initial position of the object is incorrectly detected as foreground; and (2) when an object that exists in the first frame begins to move after a few frames, the newly revealed background called “ghosts” is detected. Ghosts do not correspond to any real object in practice and have to be discarded. In this paper, we proposed an effective method of ghost detection and removal based on the SuBSENSE framework [11]. First of all, an adaptive sample-based two-layer background model, main background model, and candidate background model were proposed to remove the first kind of ghosts. The main model aimed to store the adaptive background samples. In the model update, the background samples replaced in the main model were stored in the candidate model, that is, the background samples were not actually deleted. Doing can extend the lifespan of the background samples, removing the periodic motion background and the ghosts of intermittent moving objects. Then, we utilized the histogram similarity of moving areas between the first frame and the ghost formation frame to deal with the second kind of ghosts. Furthermore, in order to make the segmentation thresholds adapt to the analyzed scenarios, the distance threshold was initialized according to background dynamics, dynamic range, and texture complexity of a scenario, and then it was updated automatically in the subsequent frames by combining spatiotemporal local information. The similarity threshold of local binary similarity pattern (LBSP) feature was computed based on the mean squared error of the background samples sets. The improved thresholds not only effectively detected camouflaged objects in low-contrast regions, but also suppressed dynamic background in high-contrast regions. In addition, since there was often a clear boundary between background and foreground area, the time subsampling factor increased in the edge to slow down the neighborhood diffusion of the background samples, retaining the static foreground for a long time. For the sake of convenience in writing, we use the abbreviation “GhostDeReBS” to represent our proposed method in the paper. An overview of GhostDeReBS’ framework is shown in Figure 1.

The rest of the paper is organized as follows. Section 2 describes our proposed algorithm in detail. The experimental evaluation is presented in Section 3. Finally, Section 4 gives the conclusions.

## 2. Methodology

We describe our proposed approach from four aspects. First, we present the sample-based two-layer background model to classify background and foreground in Section 2.1. This model not only suppressed ghosts caused by intermittent motion objects, but also reduced false positives caused by periodic motion background. Second, we show the detection and removal process of ghosts caused by incorrect model initialization based on the histogram similarity and feedback scheme in Section 2.2. Third, we describe how to update the two-layer background model in Section 2.3. Finally, we adaptively determine three important parameters according to spatial-temporal characteristics of the scene itself in Section 2.4.

### 2.1. Sample-Based Two-Layer Background Model and Background/Foreground Classification

Due to the use of the neighborhood diffusion mechanism in sample-based background subtraction, foreground objects that remain motionless for a long time are incorporated into the background. Later on, the foreground object is removed, and the initial position of the object is detected. This is because the true background samples are deleted after maintaining for a short time in the model update process. In order to retain the background samples for a long time, the methods [10,11] increased the number of background samples. However, performance degrades when the number of samples exceeds 50. The method [12] used the feature of the current observation to replace the sample with the minimum weight. However, it cannot quickly adapt environmental change.

Unlike the above methods, we presented a sample-based two-layer background model in the paper: The main model $BG_{a}(x)$ and the candidate model $BG_{c}(x)$. $BG_{a}(x)$ could adapt to scene changes. $BG_{c}(x)$ was composed of the background samples replaced in $BG_{a}(x)$. It can be seen that the lifespan of background samples was extended to $BG_{c}(x)$. Specifically, each pixel x was modeled by a set of $N_{a}$ sample values $bg_{a,k}(x)\;(k=1,2,\cdots ,N_{a})$ and a set of $N_{c}$ candidate sample values $bg_{c,k}(x)\;(k=1,2,N_{a}\cdots ,N_{c})$:

(1) $$BG_{a}(x)=\left\{bg_{a,1}(x),\;bg_{a,2}(x),\cdots ,bg_{a,N_{a}}(x)\right\}$$

(2) $$BG_{c}(x)=\left\{bg_{c,1}(x),\;bg_{c,2}(x),\cdots ,bg_{c,N_{c}}(x)\right\}$$

Similar to SuBSENSE, we also utilized color information and local binary similarity pattern (LBSP) feature to construct background model. That is, $bg_{a,k}(x)$ and $bg_{c,k}(x)$ are defined by a sixtuple:

(3) $$bg_{u,k}(x)=\left(i_{u,R,k}(x),i_{u,G,k}(x),i_{u,B,k}(x),\mathrm{intra}-LBSP_{u,R,k}(x),\mathrm{intra}-LBSP_{u,G,k}(x),\mathrm{intra}-LBSP_{u,B,k}(x)\right)$$

where u∈{a,c}, $i_{u,R,k}(x)$, $i_{u,G,k}(x)$, and $i_{u,B,k}(x)$ are the color intensity of RGB three channels at location x, respectively. $\mathrm{intra}-LBSP_{u,R,k}(x)$, $\mathrm{intra}-LBSP_{u,G,k}(x)$, and $\mathrm{intra}-LBSP_{u,B,k}(x)$ are the intra-LBSP texture feature [11], which can be defined as

(4) $$\mathrm{intra}-LBSP_{u,C,k}(x)=\sum_{p=0}^{P-1}d\left(i_{u,C,k}(p),i_{u,C,k}(x)\right).2^{p}$$

where

(5) $$d\left(i_{u,C,k}(p),i_{u,C,k}(x)\right)=\left\{ \begin{array}{l} 1,\;\mathrm{if}\;\left|i_{u,C,k}(p)-i_{u,C,k}(x)\right|>g_{u,C,k}(x)\\ 0,\;\mathrm{otherwise} \end{array}\right.$$

Here, C∈{R,G,B}, p is the neighboring pixel of x. $i_{u,C,k}(x)$ is the reference values of intra-LBSP descriptor. $i_{u,C,k}(x)$ and $i_{u,C,k}(p)$ come from the current frame. $g_{u,C,k}(x)$ is the internal similarity threshold of LBSP, which is discussed in detailed in Section 2.4.1.

It is worth mentioning, at the initial time, that $bg_{a,k}(x)$ ($k=1,2,\ldots ,N_{a}$) were randomly and independently selected from the color, and the intra-LBSP feature of 5*5 neighborhood pixels of x. $bg_{c,k}(x)$ ($k=1,2,\ldots ,N_{c}$) was set to 0. Meanwhile, similar to SuBSENSE, we also added an inter-LBSP descriptor to suppress shadow when the color values of the current frame did not match with those of background samples. The inter-LBSP feature is defined as

(6) $$\mathrm{inter}-LBSP_{u,C,k}(x)=\sum_{p=0}^{P-1}d\left(i_{u,C,k}(p),i_{u,C,k}^{\prime }(x)\right).2^{p}$$

Here, $i_{u,C,k}^{\prime }(x)$ are the reference values of the inter-LBSP descriptor, which comes from the color intensity of the background sample.

When a new input frame $I_{t}(t\geq 2)$ comes at time t, each pixel x was first classified as foreground ($f_{t}(x)=1$) or background ($f_{t}(x)=0$) by matching $I_{t}(x)$ with its respective background sample set $BG_{a}(x)$, i.e.,

(7) $$f_{t}(x)=\left\{ \begin{array}{l} 1,\;\mathrm{if}\;\#\{bg_{a,k}(x)|dist\left(I_{t}(x),bg_{a,k}(x)\right)<R(x),k=1,2,\ldots ,N_{a}{\}<\#}_{min}\\ 0,\;\mathrm{otherwise} \end{array}\right.$$

where

(8) $$I_{t}(x)=(i_{R,t}(x),i_{G,t}(x),i_{B,t}(x),\mathrm{intra}-LBSP_{R,t}(x),\mathrm{intra}-LBSP_{G,t}(x),\mathrm{intra}-LBSP_{B,t}(x))$$

(9) $$\begin{array}{l} dist\;\left(I_{t}(x),bg_{a,k}(x)\right)\\ =\left\{\underset{C\in \{R,G,B\}}{\max}\left\{\left|i_{C,t}(x)-i_{a,C,k}(x)\right|\right\},\underset{C\in \{R,G,B\}}{\max}\left\{Ham\left(\mathrm{intra}-LBSP_{C,t}(x),\mathrm{intra}-LBSP_{a,C,k}(x)\right)\right\}\right\} \end{array}$$

(10) $$R(x)=\{R_{color}(x),R_{lbsp}(x)\}$$

Here, # represents the number of elements in the collection, ${\#}_{min}$ is the minimal number of matches required for a background classification. $i_{R,t}(x)$, $i_{G,t}(x)$, and $i_{B,t}(x)$ are the color intensity at location x at time t, respectively. $\mathrm{intra}-LBSP_{R,t}(x)$, $\mathrm{intra}-LBSP_{G,t}(x)$, and $\mathrm{intra}-LBSP_{B,t}(x)$ are the intra-LBSP texture feature. dist(·,·) is a distance function between the current observation and a given background sample, which includes two elements: Color and texture distance. Ham(·,·) represents Hamming distance. R(x) is the distance threshold, which includes two elements: Color threshold $R_{color}(x)$ and LBSP texture distance threshold $R_{lbsp}(x)$. The discussion about R(x) is postponed to Section 2.4.2.

Then, if the pixel x is classified as foreground, $I_{t}(x)$ continues to compare with background model $BG_{c}(x)$. When $I_{t}(x)$ matches with $BG_{c}(x)$, it indicates that the pixel x was previously judged as background. Thus, the pixel x is considered as ghosts caused by removed foreground objects. The final output segmentation map $fg_{t}$ can be obtained by

(11) $$fg_{t}(x)=\left\{ \begin{array}{l} 0,\;\mathrm{if}\;f_{t}(x)=1\&\#\{bg_{c,k}(x)|dist\left(I_{t}(x),bg_{c,k}(x)\right)<R(x),k=1,2,\ldots ,N_{c}\}\geq {\#}_{min}\\ 0,\;\mathrm{if}\;f_{t}(x)=0\;\\ 1,\;\mathrm{if}\;f_{t}(x)=1\&\#\{bg_{c,k}(x)|dist\left(I_{t}(x),bg_{c,k}(x)\right)\geq R(x),k=1,2,\ldots ,N_{c}{\}<\#}_{min} \end{array}\right.$$

**Figure 2:**
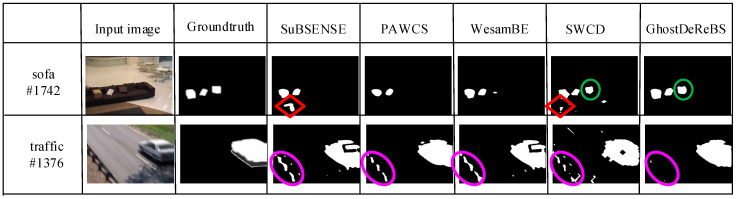
Comparison of the detection results among five methods.

Figure 2 shows the detection results of five methods, respectively, on the “sofa” #1742 and “traffic” #1376 frame from the CDnet 2014 dataset [27]. The description of this dataset is postponed to Section 3. The #1742 frame in the sofa sequence included three objects: A light-yellow box, white plastic bag, and briefcase. The light-yellow box was a static object on the floor which was then moved onto the sofa. A ghost was left on the floor (marked using red ◇) in the detection results of SuBSENSE [11] and SWCD [13]. However, our two-layer not only suppressed ghosts, but also detected camouflaged static foreground object (marked using green 〇 in Figure 2) because of the adaptive distance and LBSP threshold in Section 2.4. Moreover, the periodic motion background often happened because of the camera jitter, as shown in the traffic sequence in Figure 2, and a lot of false positives (i.e., marked using purple 〇 in Figure 2) occurred. Compared with SuBSENSE, PAWCS [16], WeSamBE [12], and SWCD, our model effectively removed these false positive detection. The reason is that the periodic background motion made the background samples appear intermittently. The earlier background samples were stored in the candidate background model in our proposed method. When Equation (11) was executed, the dynamic background could be suppressed. However, these earlier samples could have been deleted in the other methods, making the current observation unmatched with background model.

### 2.2. Detection and Removal of the Second Type of Ghost

The two-layer background model could only remove ghosts caused by the first situation and could not do anything about the second kind of ghosts mentioned in Section 1, since the newly revealed background did not match with $BG_{a}(x)$ and $BG_{c}(x)$. In order to eliminate these ghosts quickly, some literatures [10,11] have increased the neighborhood diffusion rate. However, long-term static foreground objects were also incorporated into the background. In this paper, we eliminated the second kind of ghosts based on feedback mechanism and the histogram similarity. The method was as follows.

**Figure 3:**
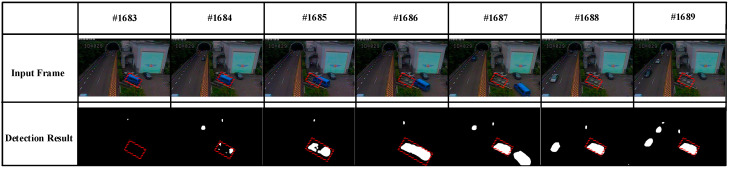
Formation process of ghosts.

**Figure 4:**
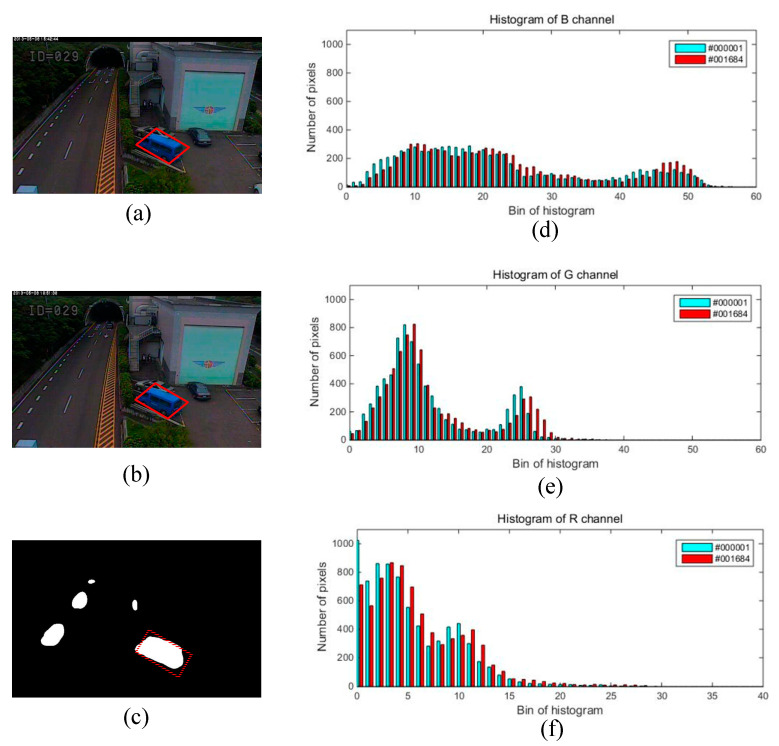
Histogram comparison of ghost region between #000001 and #001684. The region marked using red □ is the approximate location of the ghosts: (**a**) #000001; (**b**) #001684; (**c**) detection result of #001689; (**d**) histogram of B channel; (**e**) histogram of G channel; (**f**) histogram of R channel.

Taking the “tunnelExit_0_35fps” video sequence as an example as shown in Figure 3, we analyzed the formation process of ghosts caused by an object that existed in the first frame. There was a static blue minibus from #000001 to #001683 (marked using red □) in this video sequence. It started moving from #001684, and a few foreground pixels were detected in the region where the blue minibus was located. Then, foreground pixels continued to increase until the moving object and its ghosts separated, as shown in #001685, #001686, and #001687. Finally, a stable ghost region was formed in #001688 and #001689 frame (marked using red □). Of course, this process is only a necessary condition for the formation of ghosts because this situation can also occur when a normally moving object becomes motionless. However, as shown in Figure 4d–f., the histograms of the ghost region (marked using red □ in Figure 4c had high similarity on the #000001 and #001684 frames. On the contrary, the similarity of the static foreground region was often very low on the first frame and the frame appearing objects because of the difference of background and objects. Thus, we could utilize this characteristic to distinguish the two case. Here, we needed to solve the following four issues.

First, it was necessary to determine how to obtain the stable region. The connected foreground region $W_{t}(x)$, where pixel x was located at time t, was extracted by 8 neighborhood diffusion. The stable foreground region $Y_{t}(x)$ was obtained until the difference of the number of pixels located at the connected region among three adjacent frames was less than the specified threshold.

(12) $$Y_{t}(x)=\left\{y|y\in W_{t}(x)\&\frac{\left|Reg_{t-1}(x)-Reg_{t-2}(x)\right|}{\left|Reg_{t}(x)-Reg_{t-2}(x)\right|}<th_{re}\right\}$$

where $Reg_{t}(x)$ is defined as the number of pixels of the connected region. We experimentally set the threshold $th_{re}$ to 10%.

Second, it was necessary to determine how to get the frame number where the static foreground started moving. We constructed a counter FCMT to count the times that each pixel was continuously identified as a foreground pixel. Then, FN was used to record the frame number t in which each pixel x starts to move. That is, FCMT was equal to 1:

(13) $$FCMT_{t}(x)=\left\{ \begin{array}{ll} FCMT_{t}(x)+1, & \;\mathrm{if}\;fg_{t}(x)=1\\ 0, & \;\mathrm{otherwise} \end{array}\right.$$

(14) $$FN_{t}(x)=\left\{ \begin{array}{ll} t, & \;\mathrm{if}\;FCMT_{t}(x)=1\\ FN_{t}(x), & \;\mathrm{otherwise} \end{array}\right.$$

Third, it was necessary to determine how to compute the histogram similarity of the stable region between the first frame and the frame forming ghost. We used the MDPA histogram distance [28] to compute the histogram similarity of the connected regions at location x.

(15) $$MDPA_{}^{C}\left(H_{1}^{C}(x),H_{FN_{t}(x)}^{C}(x)\right)=\frac{\sum_{i=0}^{M-1}\left|\sum_{j=0}^{i}\left(H_{1}^{C}(x)[j]-H_{FN_{t}(x)}^{C}(x)[j]\right)\right|}{\sum_{i=0}^{M-1}H_{1}^{C}(x)[i]}$$

Here, C∈{R,G,B}, $H_{1}^{C}(x)$, and $H_{FN_{t}(x)}^{C}(x)$ denote the histograms of the connected region corresponding to the first frame and the frame starts to appear ghosts at location x, respectively, and the color histogram of R, G, B channels was quantified as the M (M=64) bin.

If two histograms were similar, the connected region at location x was considered as ghosts.

(16) $$G_{t}(x)=\left\{ \begin{array}{l} 1,\;\mathrm{if}\;\underset{C\in \{R,G,B\}}{\max}\left\{MDPA_{}^{C}\left(H_{1}^{C}(x),H_{FN}^{C}(x)\right)\right\}<T_{hist}\\ 0,\;\mathrm{otherwise} \end{array}\right.$$

Here, $T_{hist}$ was the histogram similarity threshold and was set to 3 experimentally in the paper.

**Figure 5:**
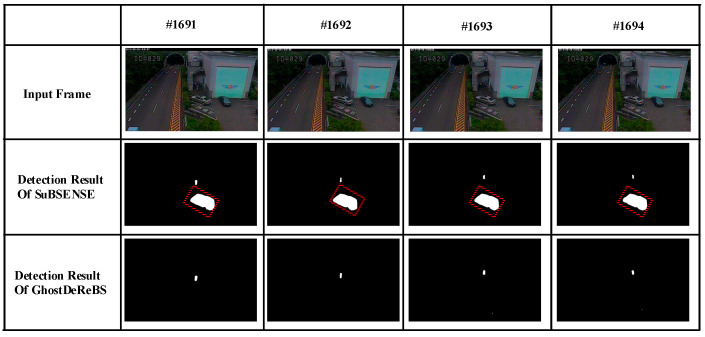
Detection result comparison of SuBSENSE and GhostDeReBS.

Finally, the ghosts were removed. When a pixel was considered a ghost ($G_{t}(x)=1$), it was classified as background, and the background model was reinitialized using the color and intra-LBSP feature of 5*5 neighborhood pixels of the current frame. As shown in Figure 5, our proposed method could effectively remove ghosts caused by incorrect model initialization, but a ghost was left (marked using red □) in the detection results of SuBSENSE.

### 2.3. Background Model Update

In complicate practical scenarios, background changes (i.e., gradual or sudden illumination change, camera jitter, PTZ) often occur. Therefore, it was necessary to update background model to adapt the scene changes after background/foreground classification. The conservative strategy was used to select the pixels which needed to be updated, and a random observation replacement policy was used to select the samples which was updated in the paper. So, the update process of our two-layer background model ($BG_{a}(x)$ and $BG_{c}(x)$) was as follows.

First, $BG_{a}(x)$ was updated using the current observation. When a new pixel x in current frame was classified as background, a randomly selected background sample $bg_{a,k}(x)$ from $BG_{a}(x)$ had 1/ϕ(x) probability to be replaced by the features $I_{t}(x)$ of the current observation x. Meanwhile, at y, a neighbor of x, a randomly picked sample $bg_{a,i}(y)$ from $BG_{a}(y)$ was also replaced by $I_{t}(x)$ with 1/ϕ(x) probability. That is,

(17) $$bg_{a,k}(x)=\left\{ \begin{array}{ll} I_{t}(x), & \;\mathrm{if}\;\mathrm{rand}(0,\phi (x))=0\\ bg_{a,k}(x), & \;\mathrm{otherwise} \end{array}\right.$$

(18) $$bg_{a,i}(y)=\left\{ \begin{array}{ll} I_{t}(x), & \;\mathrm{if}\;\mathrm{rand}(0,\phi (x))=0\\ bg_{a,i}(y), & \;\mathrm{otherwise} \end{array}\right.$$

Here, ϕ(x) is the time subsampling factor, and rand(0,ϕ(x)) is a function, which obtains a random number between 0 and ϕ(x).

Then, the candidate background model $BG_{c}(x)$ was updated by the sample replaced in $BG_{c}(a)$ Specifically, if the difference between the background sample $bg_{a,k}(x)$ replaced and $I_{t}(x)$ was larger than the distance threshold R(x), $bg_{a,k}(x)$ was stored in $BG_{c}(x)$ before it was replaced.

(19) $$bg_{c,j}(x)=\left\{ \begin{array}{l} bg_{a,k}(x),\;\mathrm{if}\;\left|bg_{a,k}(x)-I_{t}(x)\right|>R(x)\\ bg_{c,j}(x),\;\mathrm{otherwise} \end{array}\right.$$

where

(20) j=rand(0,ϕ(x))

It is worth mentioning that the conservative update strategy caused the deadlocks, and the false positive (i.e., ghosts) was difficult to eliminate, since only the pixels marked as background were updated. However, our candidate background model could solve the deadlock problem effectively because $bg_{a,k}(x)$ was not actually deleted but stored in $BG_{c}(x)$, as shown in Equation (19). The current observation may not match with the samples in $BG_{a}(x)$ when intermittent moving objects are removed, but it matched with the samples in $BG_{c}(x)$. Thus, the current observation was still classified as background, and then there were no ghosts left.

### 2.4. Parameter Analysis

As stated above, our proposed approach involved three important parameters: Similarity threshold of LBSP $g_{u,C,k}(x)$, distance threshold R(x), and time subsampling factor ϕ(x). We analyze them in detail in this section.

#### 2.4.1. Similarity Threshold of LBSP

**Figure 6:**
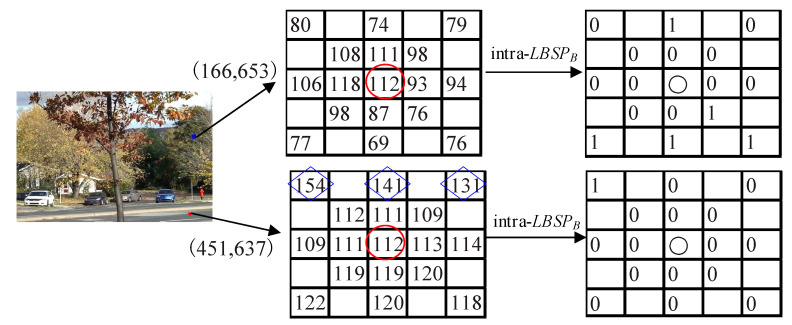
Comparison of intra-LBSP features of two pixels on blue channel. The pixel (451,637) was located in the static region and the pixel (166,653) was located in the dynamic region.

In SuBSENSE, $g_{u,C,k}(x)$ was initialized to $g_{r}.i_{u,C,k}(x)$ and $g_{r}$ was set to 0.3 experimentally. Thus, the initial value of $g_{u,C,k}(x)$ only depends on the intensity of the pixel x and is not relevant to the location of x, so it is a global threshold. It is obviously unreasonable to use the same threshold in different scenarios or different regions of the same scenario because of the same $i_{u,C,k}(x)$. This case is illustrated by the pixels (451, 637) and (166, 653) of #000001 frame from “fall” video sequence of the CDnet 2014 dataset in Figure 6. The pixel (451, 637) locates in the static region while the pixel (166, 653) locates in the dynamic region. Both of their reference intensities located in the red 〇 are 112. Thus, the thresholds of both pixels are set to 33 (0.3*112 = 33) at the initial time. In fact, the threshold of the pixel (451, 637) should be set to a smaller value (i.e. 15) for detecting the horizontal texture in “154”, “141” and “131” (marked using the blue ◇). The threshold of the pixel (166, 653) should be set to a larger value (i.e., 50) for suppressing dynamic background. Although $g_{u,C,k}(x)$ is automatically regulated over time based on the texture magnitude of the analyzed scenes in SuBSENSE, making little texture scenes with a smaller threshold than that cluttered scenes. However, the regulation is based on frame-level texture magnitude rather than pixel-level texture magnitude. Thus, $g_{u,C,k}(x)$ is always a global threshold and cannot reflect the characteristics of a pixel or a local area.

As analyzed above, $g_{u,C,k}(x)$ should vary with different scenarios or different regions. We adaptively computed $g_{u,C,k}(x)$ based on the mean squared error of the background samples in the paper. First, the background samples at location x integrated the spatial-temporal local information of the pixel, since some of them came from the feature of the previous frames and the others came from the feature of neighborhood pixel. Then, the distribution of the background sample set in high-contrast region (i.e., swaying trees, rippling water) was more dispersed than that in low-contrast regions (i.e., road, wall). Therefore, the dynamic background region had a large mean squared error (MSE). Correspondingly, a small value was produced in the static background region. Since the foreground pixels or noise can be involved into the background model in the model update, the background samples with large difference (i.e., the maximum and minimum sample values) in the model should not participate the calculation. Thus, $g_{u,C,k}(x)$ is defined as

(21) $$g_{u,C,k}(x)=h_{a,C}(x),\;k=1,2,\cdots ,N_{u}$$

where

(22) $$h_{a,C}(x)=\sqrt{\frac{\sum_{q=1}^{n_{a}}{\left(i_{a,C,q}(x)-\bar{bg_{a,C}(x)}\right)}^{2}}{n_{a}}}$$

(23) $$S_{a}=\{i_{a,C,k}(x)|i_{a,C,k}(x)-bg_{a,C,\min}(x)>th_{1}\&|bg_{a,C,\max}(x)-i_{a,C,k}(x)|>th_{1},k=1,2,\cdots ,N_{a}\}$$

(24) $$\bar{bg_{a,C}(x)}=\frac{\sum_{i_{a,C,q}(x)\in S_{a}}^{}i_{a,C,q}(x)}{n_{a}}$$

Here, $n_{a}$ is the number of background samples in set $S_{a}$ at location x. $bg_{a,C,\min}(x)$ and $bg_{a,C,\max}(x)$ are the minimum and maximum color sample values in $BG_{u}(x)$, respectively. $th_{1}$ is disturbance threshold and was set to 3 experimentally. $g_{u,C,k}(x)$ was limited to the interval [3,30]. It is worth mentioning that the main model and the candidate model utilized the same similarity thresholds in this paper, since the main model better could reflect the change of scene.

**Figure 7:**
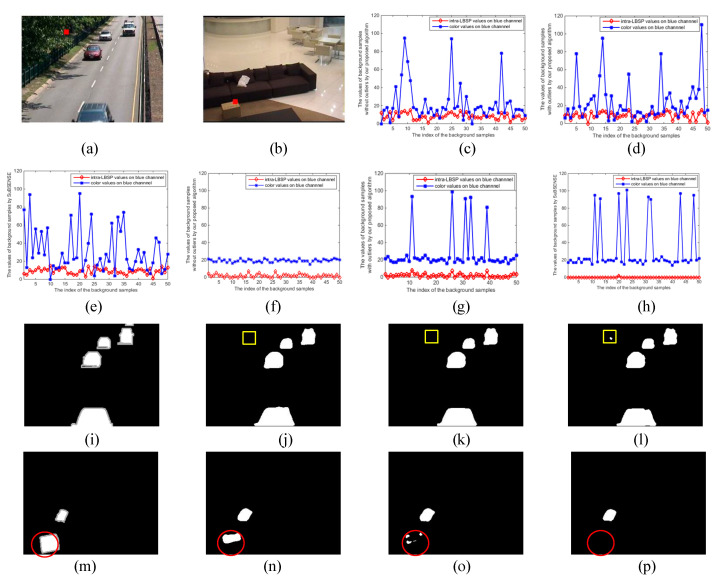
Comparison of background sample set between in static and dynamic regions. (**a**) Location of observed pixel (37, 102) on highway #001430. (**b**) Location of observed pixel (194, 73) on sofa #001030. (**c**) Background sample set obtained on (37, 102) by our proposed algorithm without outliers. (**d**) Background sample set obtained on (37, 102) by our proposed algorithm with outliers. (**e**) Background sample set obtained on (37, 102) by SuBSENSE. (**f**) Background sample set obtained on (194, 73) by our proposed algorithm without outliers. (**g**) Background sample set obtained on (194, 73) by our proposed algorithm with outliers. (**h**) Background sample set obtained on (194, 73) by SuBSENSE. (**i**) Ground-truth masks of highway #001430. (**j**) Detection results of our proposed algorithm without outliers on highway #001430. (**k**) Detection results of proposed algorithm with outliers on highway #001430. (**l**) Detection results of SuBSENSE on highway #001430. (**m**) Ground-truth masks of sofa #001030. (**n**) Detection results of our proposed algorithm without outliers on sofa #001030. (**o**) Detection results of proposed algorithm with outliers on sofa #001030. (**p**) Detection results of SuBSENSE on sofa #001030.

As an example of the pixel (37, 102) on the #001430 frame from the “highway” video sequence and the pixel (194, 73) on the #001030 frame from the “sofa” video sequence in Figure 7, Figure 7a,b show the location of two pixels in the original frames. The former locates in the dynamic region and the latter locates in the static region. Figure 7c–h are the color and intra-LBSP features in background sample set obtained on blue channel using three methods: Our proposed algorithm with and without outliers, and SuBSENSE. Here, the intra-LBSP features were defined as the number of “1.” Table 1 lists MSE of color and LBSP feature in background sample set on three methods. It is not hard to find from Figure 7f–h that our proposed method could obtain richer LBSP texture features at location (194, 73) than SuBSENSE, which can also be illustrated by a larger LBSP MSE in Table 1. This was beneficial to detect camouflaged static foreground objects. Then, we can see by comparing Figure 7d,e with Figure 7g,h that the color and intra-LBSP features at location (37, 102) were more widely distributed than those at (194, 73) using our proposed algorithm with outliers and SuBSENSE. The similarity threshold at location (37, 102) should be set to a larger value. However, the difference of the color MSEs was small at two locations. That is because some outliers (i.e., foreground pixels, noise) were included in the background sample set. It is easy to cause the camouflaged object to go missing, which can be demonstrated by the missed box in Figure 7o,p (marked by red 〇). Thus, these outliers were excluded in our final algorithm (see Equation (22)) for detecting the camouflaged object (see Figure 7n). Moreover, since the distribution of the background sample set was more disperse at location (37, 102), the difference of the color MSE without outliers or with outliers was not obvious. Therefore, outliers only had a great influence on the static flat region. In addition, although our proposed algorithm without outliers had a smaller color MSE (21.57) than that of SuBSENSE (24.55) on pixel (37, 102), the dynamic background (marked by yellow □) was still suppressed, as shown in Figure 7j. It attributes to our two-layer background model, since the periodic motion background samples were stored in the candidate model and could match with the current pixel.

#### 2.4.2. Distance Threshold

The distance threshold R(x) is an extremely important parameter which adjusts the precision and sensitivity of the background model for the local changes. In initial time, the color threshold $R_{color}(x)$ is set to 30 in SuBSENSE [11], 23 in WeSamBE [12], and 35 in SWCD [13], and the texture threshold $R_{lbsp}(x)$ is set to 3 for all the experimental scenarios. Then, they are automatically adjusted over time based on the historical detection results of each pixel. In fact, the initial value $R_{color}^{0}(x)$ and $R_{lbsp}^{0}(x)$ directly influences the final segmentation results. It is unreasonable that they are set to the same value for all scenes. Instead, they should be set to a large value in these scenes with highly dynamic background and rich texture information for reducing false negatives. Otherwise, it is a small value in static and weak texture scenes for increasing the true positive. In the paper, we initialized this parameter according to the dynamic range, background dynamics, and texture complexity of every scene. In fact, the dynamic range of a scene reflects the distribution of pixels. A narrow dynamic range means that the distribution of pixels relatively concentrates and the difference between pixels is small in the scene. Thus, a small distance threshold should be selected so as to detect the moving objects in the scene. The background dynamics represent the background changes. These changes could be local (i.e., swaying tree in the wind) or global (i.e., camera motion). In order to make sure that changing background is not detected as foreground, a large distance threshold should be set. A rich texture can improve identification of the foreground, but it increases the false positive in background region with complex texture. This is a tradeoff. Thus, we appropriately increased the distance threshold in the texture region.

First, the distribution of the color histogram was used to measure the dynamic range of an image in Equation (25). If most of color values of an image are concentrated on a few bins, the dynamic range is small.

(25) $$RI=(X_{L}+(1-K_{L}))+(X_{a}+(1-K_{a}))+(X_{b}+(1-K_{b}))$$

where

(26) $$K_{cc}=\frac{\sum_{H_{cc}(i)>2(h*w)/256}^{}H_{cc}(i)}{\left(h*w\right)}$$

(27) $$X_{cc}=\#\{H_{cc}(i)|H_{cc}(i)\geq 2*(h*w)/256,i=1,2,\ldots ,256\}/256$$

Here, h and w specify the height and width of input image, respectively, and $H_{cc}(i)(i=0,1,2,\ldots ,255,\;\mathrm{cc}=\{L,a,b\})$ is defined as the frequency of the ith grayscale on Lab color space. $K_{cc}$ and $X_{cc}$ represent the proportion of pixels and number of grayscale with higher frequencies, respectively. Thereby, the larger $K_{cc}$ is and the smaller $X_{cc}$ is, the more concentrated the distribution pixel values are. RI effectively reflects the dynamic range of a scene, and its value is in the interval [0.3,1.3] in most of the scenarios.

Second, the change of background is measured by the mean value of the absolute difference $M_{0}$ between the first two frames. Generally, there are few moving objects in the first two frames. Thus, $M_{0}$ represents the background dynamics of the scene. $M_{0}$ is almost equal to 0 in a static scenario and a large value if the dynamic background elements are included in the scene or the camera moves.

(28) $$M_{0}=\frac{\sum_{q=1}^{h*w}\left|In_{2}(q)-In_{1}(q)\right|}{h*w}$$

Here, $In_{1}$ and $In_{2}$ are the intensity of the first and second frames, respectively.

Third, the texture complexity of the scene is measured utilizing the mean value of Laplacian texture feature of the first frame $L_{0}$. A large $L_{0}$ indicates that the scene has a strong texture.

(29) $$L_{0}=Mean\left(Laplacian(In_{1})\right)$$

where $L_{0}\in [10,60]$ in the CDnet2014 dataset.

Finally, the initial distance threshold can be obtained by

(30) $$R_{color}^{0}(x)=K_{0}*(1+RI)+M_{0}+L_{0}/a$$

(31) $$R_{lbsp}^{0}(x)=L_{0}/b$$

Here, $K_{0}$, a, and b are user-defined parameters. Comprehensively considering the research results described by the authors of [11,12,13], we bound $R_{color}^{0}(x)$ and $R_{lbsp}^{0}(x)$ to the intervals [10,40] and [1,6], respectively, to adapt most of the practical environments. Thus, we defined $K_{0}=10$, a=10, and b=10 in the paper. The modified initial R(x) can achieve a robust detection result against the environment changes.

Next, the distance thresholds need to be updated for adapting gradual background changes in test frames after their background/foreground segmentation. In Reference [11], it was adjusted according to two important indexes: Background dynamics and local segmentation noise levels.

Background dynamics of the pixel x at time t is measured by a recursive moving average $D_{\min}(x)$:

(32) $$D_{\min}(x)=D_{\min}(x)\cdot (1-\alpha )+d_{t}(x)\cdot \alpha$$

where

(33) $$d_{t}(x)=\min\{dist\left(I_{t}(x),bg_{a,k}(x)\right)|k=1,2,\cdots ,N_{a}\}$$

Here, α is the learning rate, and $\alpha^{ST}$ (=1/25) and $\alpha^{LT}$ (=1/100) are a short-term learning rate and a long-term learning rate, respectively, in SuBSENSE. $d_{t}(x)$ is the minimal normalized color-LBSP distance between all samples in $BG_{a}(x)$ and $I_{t}(x)$. Therefore, $D_{\min}(x)\approx 0$ in a completely static background region, and $D_{\min}(x)\approx 1$ in a dynamic region and foreground object region.

The local segmentation noise level is measured by the accumulator v(x) of blinking pixels (alternatively marked as foreground and background in time).

(34) $$v(x)=\left\{ \begin{array}{l} v(x)+v_{incr},{\;\mathrm{if}\;X}_{t}(x)=1\\ v(x)-v_{decr},\;\mathrm{otherwise} \end{array}\right.$$

where

(35) $$X_{t}(x)=fg_{t}(x)⊕fg_{t-1}(x)$$

Here, ⊕ refers to an XOR operation, and the increment parameter $v_{incr}$ and decrement parameter $v_{decr}$ are 1 and 0.1, respectively. v(x) converges to 0 for a stable pixel, and v(x) would have large positive for constantly changing pixels.

Based on $D_{\min}(x)$ and v(x), the distance threshold factor r(x) and distance threshold R(x) was updated for each new frame according to Equations (36)–(38). Unlike Reference [11], we fused the similarity threshold of LBSP $h_{a,C}(x)$ to update $R_{color}(x)$. The improved distance threshold could quickly respond to the change of the environment and accelerate the convergence of the algorithm, since $h_{a,C}(x)$ has a large value in dynamic background region and a small value in static background region.

(36) $$r(x)=\left\{ \begin{array}{ll} r(x)+v(x), & \;\mathrm{if}\;r(x)<{\left(1+D_{\min}(x)\cdot 2\right)}^{2}\\ r(x)-1/v(x), & \;\mathrm{otherwise} \end{array}\right.$$

(37) $$R_{color}(x)=(1-\beta )\cdot r(x)\cdot R_{color}^{0}(x)+\beta \cdot \left(h_{a,R}(x)+h_{a,G}(x)+h_{a,B}(x)\right)/3$$

(38) $$R_{lbsp}(x)=2^{r(x)}+R_{lbsp}^{0}(x)$$

where r(x) was initialized to 1, β was weighed and set to a little value (0.1 in the paper). $h_{a,R}(x)$, $h_{a,G}(x)$, and $h_{a,B}(x)$ were updated by Equation (22).

#### 2.4.3. Time Subsampling Factor

The time subsampling factor ϕ(x) is another important parameter in the sample-based detection algorithm. ϕ(x) controls the update speed of the background model. A small ϕ(x) makes the model updated with high chances, which leads to the cases in which slowly moving objects are assimilated into the background, generating false negatives. Conversely, a large ϕ(x) causes the background model to adapt to the background changes slowly, resulting in ghosts that are not eliminated for a long time, generating false positives.

ϕ(x) was initialized to 2 and was limited to the interval [2,∞]. It was updated by

(39) $$\phi (x)=\left\{ \begin{array}{ll} \phi (x)+\frac{1}{v(x)\cdot D_{\min}(x)}, & \;\mathrm{if}\;fg_{t}(x)=1\\ \phi (x)-\frac{v(x)}{D_{\min}(x)}, & \;\mathrm{otherwise} \end{array}\right.$$

Meanwhile, in order to avoid camouflaged foreground pixels into background model, the edge information was utilized to regulate the time sub-sampling factor in the process of neighbor diffusion. More precisely, there is often strong texture information at the border between the background and foreground regions. The neighborhood diffusion should slow down at the boundary. That is, we used a large $\phi^{\prime }(y)$ to update the background model of pixel y (a neighbor of x) when a pixel x was classified as background ($fg_{t}(x)=0$) and the Laplacian texture feature L(y) was larger than a user-defined threshold $th_{2}$, that is,

(40) $$\phi^{\prime }(y)=\left\{ \begin{array}{ll} \phi (x), & \;\mathrm{if}\;fg_{t}(x)=0\&L(y)<th_{2}\\ m_{0}*\phi (x), & \;\mathrm{if}\;fg_{t}(x)=0\&L(y)\geq th_{2} \end{array}\right.$$

where

(41) $$th_{2}=2\left(h_{a,R}(x)+h_{a,G}(x)+h_{a,B}(x)\right)/3$$

Here, $m_{0}$ should choose a slightly large value for slowing diffusion. The value was set to 5 experimentally in the paper.

## 3. Experimental Analysis

### 3.1. Dataset and Evaluation Metrics

In order to evaluate the performance of the proposed method, we selected the CDnet dataset [27] as the test dataset. Compared with other dataset (i.e., Wallflower, PETS), the CDnet dataset has two merits. One is the variety of scenarios. The earlier vision (CDnet 2012 dataset) offers 31 real world scenes (more than 88,000 frames) and is classified into six video categories: Baseline, camera jitter (CJ), dynamic background (DB), intermittent object motion (IOM), shadow, and thermal. In 2014, the dataset was expanded to 53 videos (nearly 160,000 frames, 11 categories). The new added 22 videos are divided into five categories: Bad weather (BW), low frame rate (LF), night videos (NV), PTZ, and turbulence, which have greater challenges. The expanded dataset almost covers all challenges on change detection. The other is labeled ground-truth masks, making the results more reasonable for comparing the proposed method with other methods.

The results were compared and quantified by the following seven metrics [27]: Recall (Re), Specificity (Sp), False Positive Rate (FPR), False Negative Rate (FNR), Percentage of Wrong Classifications (PWC), Precision (Pr), and F-Measure. The seven metrics are considered as official metrics to test the effectiveness of the change-detection algorithms.

### 3.2. Discussion

#### 3.2.1. How to Determine $N_{a}$ and $N_{c}$

We introduced two important parameters in the two-layer background model in Section 2.1: $N_{a}$ (number of background samples in the main model) and $N_{c}$ (number of candidate background samples). $N_{a}$ was set to 50, 25, and 35, respectively, in SuBSENSE, WeSamBE, and SWCD. $N_{c}$ was the parameter increased in the paper. To determine the two parameters, the relationship among $N_{a}$, $N_{c}$ and the average F-measure was analyzed on the CDnet 2014 dataset. We discussed $N_{a}$ and $N_{c}$ in interval [0,50] with the increment of 5 to remain consistent with the above-mentioned literature. Here, we only list the results of the bad weather, camera jitter, dynamic background, and low frame rate categories in Table 2, Table 3, Table 4 and Table 5, since the four categories represent four typical scenarios. For example, a bad weather scene with lots of noise and a narrow dynamic range, camera jitter with the periodic and a global background motion, dynamic background with a local background motion, and low frame rate scene with the large displacement of a moving object.

The blue entries indicate the better results in Table 2, Table 3, Table 4 and Table 5. The F-Measure score first improved and then degraded as $N_{a}$ or $N_{c}$ increased. Therefore, it is not always better for $N_{a}$ or $N_{c}$ to choose a bigger value. Too many background samples cause the model to be overfitted. By comparing $N_{c}=0$ and $N_{c}\neq 0$ in Table 2, Table 3, Table 4 and Table 5, it is not difficult to find that the candidate background model could improve the F-Measure score. Taking the camera jitter category as an example, the F-Measure score of $N_{a}=20$ and $N_{c}=10$ was improved by about 2.3% over $N_{a}=30$ and $N_{c}=0$. For the scenes with background motion, such as camera jitter and dynamic background, a large $N_{a}$ was required to store diverse background samples, and a relatively small $N_{c}$ was set to rapidly adapt to the changing scene. For example, the optimal F-Measure score was concentrated in $N_{a}=45$ and $N_{c}=25$ in Table 2 and Table 3. Moreover, it can be seen from Table 4 that $N_{a}$ was a small value in the relatively static scene and $N_{c}$ should be selected a larger value for adapting slowly changing background and remaining background samples for a long time for reuse. For example, the F-Measure score was better when $N_{a}=15$ and $N_{c}=40$ in low frame rate category. In addition, a small $N_{c}$ was needed in the scene with narrow dynamic range as shown in Table 5 because the difference among pixels was not obvious. As a consequence, $N_{a}$ and $N_{c}$ were determined according to background dynamics and the dynamic range of the scene. The computing equations are as follows.

(42) $$N_{a}(x)=m_{1}+R_{dy}(x)*m_{2}$$

(43) $$N_{c}(x)=\left(1-R_{dy}(x)\right)*m_{3}+\left(RI-0.5\right)*m_{4}$$

where

(44) $$R_{dy}(x)=\frac{\sum_{q\in W_{p}(x)}^{}f_{of}(q)}{p*p}$$

Here, $W_{p}(x)$ is the p∗p neighboring pixel of x and p=21 in the paper, $f_{of}(q)$ is the detection result of the second frame image using optical flow method, and $R_{dy}(x)$ reflects the strength of the background motion. The value of $R_{dy}(x)$ was in the interval [0,0.5] in most of the scenarios. According Table 2, Table 3, Table 4 and Table 5, it can be seen that the performance was better when $15\leq N_{a}\leq 40$ and $5\leq N_{c}\leq 50$. Thus, we set $m_{1}=10$, $m_{2}=50$, $m_{3}=10$, and $m_{4}=50$.

#### 3.2.2. Threshold Performance Analysis

In order to analyze the effect of the improved threshold in the proposed method, we list the detection results of the following four cases in Table 6: (1) Using the distance threshold, LBSP similarity threshold, and time subsampling factor in SuBSENSE; (2) using the LBSP similarity threshold and time subsampling factor in SuBSENSE and using the improved distance threshold; (3) using time subsampling factor in SuBSENSE and using the improved distance threshold and LBSP similarity threshold; and (4) using the improved distance threshold, LBSP similarity threshold, and time subsampling factor.

It can be seen from Table 6 that Re and the average F-measure score was continuously improved from case (1) to case (4). Meanwhile, by comparing case (1) and (2) and case (2) and (3) in Table 6, it is not hard to find that the average F-measure had a great improvement after using the improved LBSP similarity threshold or the improved distance threshold. The reason was that the two thresholds were adaptively determined according to the change of the scene and region itself, that is, a large threshold in the regions with a fast-changing background, and a small threshold in the static and low-contrast regions. In addition, the average F-measure had not been obviously improved after using the improved time subsampling factor, as shown in case (3) and (4), because the time subsampling factor was slightly adjusted.

### 3.3. Experimental Results

#### 3.3.1. Ghost Removal

**Figure 8:**
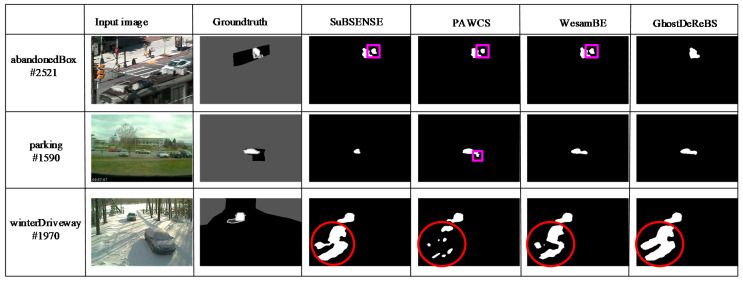
Analysis of advantages and disadvantages of our proposed method in ghost removal.

The critical ghosts appeared in intermittent object motion (IOM) category and in “tunnelExit_0_35fps” video sequence of “low framerate” category on the CDnet2014 Dataset. In Section 2, we discuss the “sofa” and “tunnelExit_0_35fps” video sequence in Figure 2 and Figure 3. It can be seen that our proposed method removed ghosts. In order to analyze the advantages and disadvantages of our proposed method, we tested more video sequences in Figure 8, which included the “abandonedBox,” ”parking,” and “winterDriveway” scenario in the IOM category. In the “abandonedBox” scenario, there was a red box on the road from the first frame, and then it started to move from the #2446 frame. In the “parking” scenario, there was a white car in the parking lot from the first frame and then it was driven away from the #1334 frame. Both the red box and car in the two video sequences were used to model the background, hence ghosts often occur (marked by pink □) after being moved away in most of detection method. However, our proposed algorithm effectively removed ghosts. It is well known that no algorithm is omnipotent. Our proposed method could not remove ghosts when the background changed, as shown in “winterDriveway” scenario in Figure 8. In future work, we will extend our method to adapt to background changes.

#### 3.3.2. Average Performance on CDnet2014 Dataset

In this section, we demonstrate the effectiveness of our proposed method by comparing Re, Sp, FPR, FNR, PWC, Pr, and F-Measure with those of the state-of-the-art change detection approaches. The website on changedetection.net (CDnet) reported detailed segmentation results and evaluation data of dozens of change-detection algorithms on CDnet. In this paper, we systematically compared the proposed method with several related the state-of-the-art methods, such as SuBSENSE, PAWCS, WeSamBE, SWCD, SharedModel [6], and BSUV-Net [18]. First, the average performance of several algorithms is summarized in Table 7. By observing Table 7, we can see that our proposed method had the highest recall (0.8456) and the lowest FNR (0.1544). In particular, the recall obtained by our method outperformed the second-best method (SuBSENSE) by about 3.3%. The precise achieved by our method was ranked second. Meanwhile, the F-measure value (0.7898) was improved by 3% compared to SWCD and was even better than BSUV-Net. Thus, the proposed method was in competition with best methods.

Furthermore, the F-measure of each category is presented in Table 8. The proposed method gave superior results in the bad weather, camera jitter, intermittent object motion, low framerate, and turbulence categories. Especially, the F-measure of the camera jitter, low framerate, and turbulence categories increased about 4.3%, 5.5%, and 11% compared to the second-best method, respectively. For the dynamic background and shadow categories, our method ranked second. However, our method was not good at the night video and PTZ categories. The reason was that the proposed algorithm could only deal with background movements in a small range.

**Figure 9:**
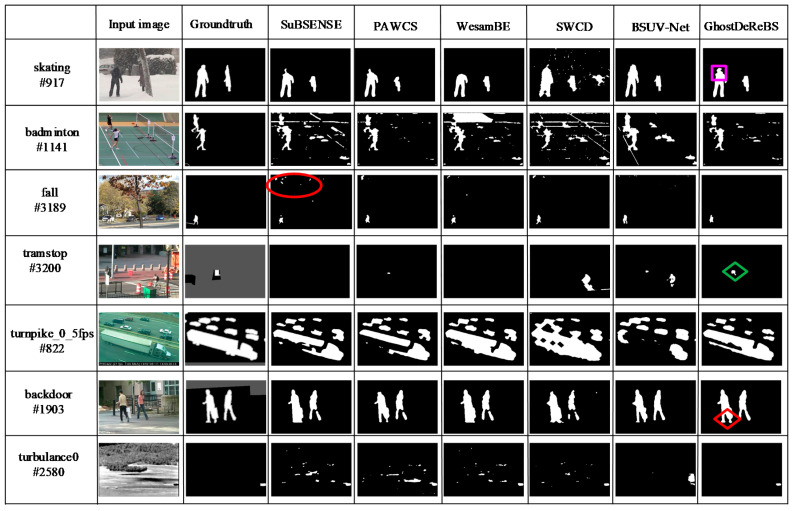
Some segmentation results for various video sequences on the CDnet 2014 dataset.

Finally, some visual results for various video sequences are shown in Figure 9. From top to bottom, the sequences are skating, badminton, fall, tramstop, turnpike_0_5fps, backdoor, and turbulance0. They are from the bad weather, camera jitter, dynamic background, intermittent object motion, low frame rate, shadow, and turbulence category, respectively. From the #1141 frame in the badminton sequence, the #3189 frame in the fall sequence, and the #2580 frame in the turbulance0 sequence, we can see that our proposed algorithm was less sensitive to dynamic background, camera jitter (periodic motion background), and noise compared with other methods because of a large distance threshold. In the “tramstop” scenario, there was a red box, which was put on the road from the #1030 frame and then kept motionless. Sample-based background subtraction often makes the static objects slowly incorporated into the background. However, our proposed algorithm still remained a part of foreground object after 2000 frames, as shown in green ◇. Meanwhile, as seen from the #917 frame in the skating sequence, our proposed algorithm detected the camouflaged person (marked using pink □). In addition, our proposed algorithm eliminated weak shadow (marked using red ◇). Therefore, our proposed algorithm was effective for suppressing dynamic background, removing the ghosts, and detecting camouflaged objects.

#### 3.3.3. Processing Speed

In this paper, all algorithms ran on an AMD Ryzen 3.7 GHz processor, which produced by AMD (Advanced Micro Devices, Inc.) in Sunnyvale, California, USA and was sourced in Chengdu, China. The experimental code is edited and complied in VS2015 and Opencv 3.0 with 16GB RAM. Table 9 lists the runtime of our proposed algorithm and SuBSENSE on three video sequences with different resolution. The runtime of our proposed algorithm was slower than that of SuBSENSE by about one-fifth on “highway,” one-fifth on “skating,” and one-third on “fall,” respectively. The reason is that Equation (12) and Equation (22) in our algorithm needed spend more time. The time complexity on “fall” was higher than “highway” and “skating” because of a higher number of background samples used on “fall.” In the future work, we will modify the update mechanism in Equation (22) and the connected region computation strategy to reduce the overall computing time of the algorithm.

## 4. Conclusions

In this paper, we proposed a ghost detection and removal method using sample-based two-layer background model and histogram similarity, which removed the ghosts caused by incorrect model initialization and intermittent object motion. In addition, the candidate background model added could decrease the false positive detection cause by periodic background motion because it extends the lifespan of the background samples. Then, we modified the color and texture distance threshold, internal similarity threshold of LBSP feature, and time subsampling factor according to the characteristic of the scene and region. The improved parameters were beneficial to suppress dynamic background and detect camouflaged objects. Our proposed algorithm was proved to be effective in comparison to other the state-of-the-art methods. However, our proposed algorithm was not suitable for the scenarios with substantial background changes. In this case, ghosts were removed slowly by the neighborhood diffusion strategy in the SuBSENSE framework. This problem is a promising direction for future work.

## Figures and Tables

**Table 1:** MSE comparison of color and LBSP feature in background sample set on three methods.

Method	SuBSENSE	Our Proposed Algorithm with Outliers	Our Proposed Algorithm without Outliers (GhostDeReBS)
highway	color: 24.55 LBSP: 3.16	color: 23.36 LBSP: 3.79	color: 21.57 LBSP: 3.32
sofa	color: 28.21 LBSP: 0.28	color: 21.79 LBSP: 1.93	color: 1.64 LBSP: 1.79

**Table 2:** Average F-Measure scores obtained on camera jitter category using different $N_{a}$ and $N_{c}$.

	$N_{c}$	0	5	10	15	20	25	30	35	40	45	50
$N_{a}$												
5		0.7046	0.7653	0.7679	0.7662	0.7660	0.7718	0.7719	0.7735	0.7725	0.7753	0.7778
10		0.7715	0.8144	0.8239	0.8302	0.8251	0.8300	0.8313	0.8332	0.8329	0.8352	0.8371
15		0.8027	0.8400	0.8497	0.8496	0.8519	0.8508	0.8522	0.8538	0.8529	0.8530	0.8524
20		0.8190	0.8484	0.8560	0.8537	0.8577	0.8589	0.8586	0.8601	0.8598	0.8589	0.8577
25		0.8277	0.8533	0.8541	0.8586	0.8582	0.8586	0.8603	0.8613	0.8610	0.8615	0.8619
30		0.8335	0.8521	0.8581	0.8599	0.8625	0.8615	0.8619	0.8625	0.8647	0.8634	0.8638
35		0.8417	0.8587	0.8605	0.8609	0.8620	0.8641	0.8631	0.8627	0.8623	0.8658	0.8629
40		0.8441	0.8575	0.8602	0.8634	0.8615	0.8626	0.8635	0.8645	0.8627	0.8643	0.8630
45		0.8442	0.8566	0.8601	0.8584	**0.8657**	0.8644	0.8631	0.8597	0.8577	0.8640	0.8598
50		0.8430	0.8489	0.8518	0.8435	0.8530	0.8535	0.8570	0.8544	0.8549	0.8558	0.8543

**Table 3:** Average F-Measure scores obtained on dynamic background category using different $N_{a}$ and $N_{c}$.

	$N_{c}$	0	5	10	15	20	25	30	35	40	45	50
$N_{a}$												
5		0.7505	0.7723	0.7911	0.8019	0.8146	0.8235	0.8292	0.8308	0.8340	0.8319	0.8313
10		0.7812	0.8041	0.8253	0.8496	0.8592	0.8644	0.8657	0.8679	0.8658	0.8611	0.8613
15		0.7995	0.8231	0.8558	0.8714	0.8782	0.8783	0.8786	0.8803	0.8750	0.8755	0.8702
20		0.8095	0.8398	0.8676	0.8800	0.8832	0.8851	0.8852	0.8845	0.8820	0.8820	0.8800
25		0.8252	0.8529	0.8797	0.8848	0.8873	0.8873	0.8861	0.8866	0.8862	0.8844	0.8836
30		0.8448	0.8667	0.8825	0.8874	0.8900	0.8897	0.8904	0.8911	0.8891	0.8886	0.8861
35		0.8589	0.8757	0.8861	0.8895	0.8922	0.8921	0.8921	0.8891	0.8898	0.8853	0.8837
40		0.8685	0.8799	0.8859	0.8926	0.8927	0.8935	0.8919	0.8910	0.8904	0.8850	0.8855
45		0.8754	0.8846	0.8894	0.8931	0.8922	**0.8940**	0.8923	0.8909	0.8891	0.8868	0.8843
50		0.8756	0.8867	0.8912	0.8922	0.8945	0.8923	0.8940	0.8900	0.8895	0.8877	0.8854

**Table 4:** Average F-Measure scores obtained on low frame rate category using different $N_{a}$ and $N_{c}$.

	$N_{c}$	0	5	10	15	20	25	30	35	40	45	50
$N_{a}$												
5		0.6487	0.6965	0.6885	0.6993	0.7162	0.7432	0.7631	0.7729	0.7793	0.7888	0.7925
10		0.6374	0.6513	0.6569	0.7040	0.7235	0.7420	0.7548	0.7643	0.7801	0.7941	0.8025
15		0.6932	0.6956	0.7007	0.7103	0.7325	0.7485	0.7511	0.7782	0.7965	0.8003	0.8085
20		0.7030	0.7003	0.7039	0.7101	0.7210	0.7414	0.7516	0.7800	0.7848	0.8064	0.8020
25		0.7071	0.7020	0.7053	0.7078	0.7252	0.7402	0.7530	0.7722	0.7937	0.7984	0.7978
30		0.7119	0.7159	0.7198	0.7079	0.7406	0.7319	0.7811	0.7927	0.7838	0.7983	0.8056
35		0.7067	0.7101	0.7170	0.7064	0.7159	0.7396	0.7599	0.7870	0.7857	0.7946	0.7985
40		0.7189	0.7138	0.7218	0.7169	0.7213	0.7402	0.7658	0.7646	0.7914	0.7965	0.8009
45		0.7149	0.7017	0.7150	0.7182	0.7258	0.7495	0.7751	0.7791	0.7817	0.7954	0.7977
50		0.7053	0.7209	0.7203	0.7229	0.7228	0.7376	0.7661	0.7769	0.7924	0.7935	0.7979

**Table 5:** Average F-Measure scores obtained on bad weather category using different $N_{a}$ and $N_{c}$.

	$N_{c}$	0	5	10	15	20	25	30	35	40	45	50
$N_{a}$												
5		0.8405	0.8495	0.8524	0.8529	0.8540	0.8535	0.8534	0.8528	0.8527	0.8528	0.8521
10		0.8628	0.8684	0.8692	0.8690	0.8681	0.8668	0.8666	0.8657	0.8639	0.8636	0.8636
15		0.8734	0.8762	0.8751	0.8743	0.8734	0.8722	0.8714	0.8704	0.8696	0.8688	0.8675
20		0.8776	0.8796	0.8792	0.8769	0.8750	0.8744	0.8731	0.8723	0.8715	0.8706	0.8694
25		0.8807	0.8828	0.8819	0.8811	0.8793	0.8775	0.8767	0.8757	0.8745	0.8739	0.8732
30		0.8840	0.8667	0.8852	0.8845	0.8828	0.8815	0.8795	0.8787	0.8771	0.8762	0.8749
35		0.8857	0.8869	0.8853	0.8830	0.8813	0.8813	0.8801	0.8789	0.8766	0.8773	0.8760
40		0.8865	0.8879	0.8861	0.8841	0.8829	0.8809	0.8791	0.8785	0.8775	0.8756	0.8750
45		0.8886	0.8883	0.8871	0.8845	0.8826	0.8808	0.8786	0.8774	0.8770	0.8751	0.8750
50		0.8888	**0.8893**	0.8872	0.8850	0.8823	0.8800	0.8779	0.8760	0.8748	0.8742	0.8733

**Table 6:** Performance analysis of four cases on CDnet2014 Dataset.

Method	Re	Sp	FPR	FNR	PWC	Pr	F-Measure
(1)	0.7569	**0.9930**	**0.0070**	0.2431	1.4777	**0.8177**	0.7535
(2)	0.7752	**0.9944**	**0.0056**	0.2248	**1.2644**	**0.8172**	0.7681
(3)	**0.8420**	0.9914	0.0086	**0.1580**	**1.3260**	0.7920	**0.7890**
(4)	**0.8456**	0.9908	0.0091	**0.1544**	1.3704	0.7892	**0.7898**

Note that red-bold entries indicate the best results in a given column, and blue-bold entries indicate that the second-best results.

**Table 7:** Overall performance analysis of several the state-of-the-art methods on CDnet2014 Dataset.

Method	Re	Sp	FPR	FNR	PWC	Pr	F-Measure
SuBSENSE	**0.8124**	0.9904	0.0096	0.1876	1.6780	0.7509	0.7408
PAWCS	0.7718	**0.9949**	**0.0051**	0.2282	**1.1992**	0.7857	0.7403
SharedModel	0.8098	0.9912	0.0088	0.1902	1.4996	0.7503	0.7474
WeSamBE	0.7955	0.9924	0.0076	0.2045	1.5105	0.7679	0.7446
SWCD	0.7839	0.9930	0.0070	0.2161	1.3414	0.7527	0.7583
BSUV-Net	0.8203	**0.9946**	**0.0054**	**0.1797**	**1.1402**	**0.8113**	**0.7868**
GhostDeReBS	**0.8456**	0.9908	0.0092	**0.1544**	1.3704	**0.7892**	**0.7898**

Note that red-bold entries indicate the best results in a given column, and blue-bold entries indicate that the second-best results

**Table 8:** F-measure comparison of each category on the CDnet 2014 dataset.

Category	SuBSENSE	PAWCS	SharedModel	WeSamBE	SWCD	BSUV-Net	GhostDeReBS
BW	0.8619	0.8152	0.8480	0.8608	0.8233	**0.8713**	**0.8718**
baseline	0.9503	0.9397	**0.9522**	0.9413	0.9214	**0.9693**	0.9517
CJ	**0.8152**	0.8137	0.8141	0.7976	0.7411	0.7743	**0.8583**
DB	0.8177	**0.8938**	0.8222	0.7440	0.8645	0.7967	**0.8817**
IOM	0.6569	**0.7764**	0.6727	0.7392	0.7092	0.7499	**0.7841**
LF	0.6445	0.6588	0.7286	0.6602	**0.7374**	0.6797	**0.7923**
NV	0.5599	0.4152	0.5419	**0.5929**	0.5807	**0.6987**	0.5141
PTZ	0.3476	**0.4615**	0.3860	0.3844	0.4545	**0.6282**	0.4112
shadow	0.8986	0.8913	0.8898	0.8999	0.8779	**0.9233**	**0.9131**
thermal	0.8171	**0.8324**	0.8319	0.7962	**0.8581**	**0.8581**	0.8190
turbulence	**0.7792**	0.6450	0.7339	0.7737	0.7735	0.7051	**0.8907**

Note that red-bold entries indicate the best results in a given row, and blue-bold entries indicate that the second-best results.

**Table 9:** Runtime comparison on three video sequences with different resolution.

Method	Highway (240*320)	Skating (360*540)	Fall (480*720)
SuBSENSE	15.3 fps	6.7 fps	4.2 fps
GhostDeReBS	12.6 fps	5.2 fps	2.8fps
